# Artificial Intelligence in Gastric Cancer: Identifying Gastric Cancer Using Endoscopic Images with Convolutional Neural Network

**DOI:** 10.3390/cancers13215253

**Published:** 2021-10-20

**Authors:** Md. Mohaimenul Islam, Tahmina Nasrin Poly, Bruno Andreas Walther, Ming-Chin Lin, Yu-Chuan (Jack) Li

**Affiliations:** 1 Graduate Institute of Biomedical Informatics, College of Medical Science and Technology, Taipei Medical University, Taipei 110, Taiwan; d610106004@tmu.edu.tw (M.M.I.); d610108004@tmu.edu.tw (T.N.P.); arbiter@tmu.edu.tw (M.-C.L.); 2 International Center for Health Information Technology (ICHIT), Taipei Medical University, Taipei 110, Taiwan; 3 Research Center of Big Data and Meta-Analysis, Wan Fang Hospital, Taipei Medical University, Taipei 116, Taiwan; 4 Deep Sea Ecology and Technology, Alfred-Wegener-Institut Helmholtz-Zentrum für Polar- und Meeresforschung, Am Handelshafen 12, D-27570 Bremerhaven, Germany; bwalther@awi.de; 5 Professional Master Program in Artificial Intelligence in Medicine, Taipei Medical University, Taipei 110, Taiwan; 6 Research Center for Artificial Intelligence in Medicine, Taipei Medical University, Taipei 110, Taiwan

**Keywords:** convolutional neural network, deep learning, gastric cancer, endoscopy image, artificial intelligence

## Abstract

**Simple Summary:**

Gastric cancer (GC) is one of the most newly diagnosed cancers and the fifth leading cause of death globally. Previous studies reported that the detection rate of gastric cancer (EGC) at an earlier stage is low, and the overall false-negative rate with esophagogastroduodenoscopy (EGD) is up to 25.8%, which often leads to inappropriate treatment. Accurate diagnosis of EGC can reduce unnecessary interventions and benefits treatment planning. Convolutional neural network (CNN) models have recently shown promising performance in analyzing medical images, including endoscopy. This study shows that an automated tool based on the CNN model could improve EGC diagnosis and treatment decision.

**Abstract:**

Gastric cancer (GC) is one of the most newly diagnosed cancers and the fifth leading cause of death globally. Identification of early gastric cancer (EGC) can ensure quick treatment and reduce significant mortality. Therefore, we aimed to conduct a systematic review with a meta-analysis of current literature to evaluate the performance of the CNN model in detecting EGC. We conducted a systematic search in the online databases (e.g., PubMed, Embase, and Web of Science) for all relevant original studies on the subject of CNN in EGC published between 1 January 2010, and 26 March 2021. The Quality Assessment of Diagnostic Accuracy Studies-2 was used to assess the risk of bias. Pooled sensitivity, specificity, positive likelihood ratio, negative likelihood ratio, and diagnostic odds ratio were calculated. Moreover, a summary receiver operating characteristic curve (SROC) was plotted. Of the 171 studies retrieved, 15 studies met inclusion criteria. The application of the CNN model in the diagnosis of EGC achieved a SROC of 0.95, with corresponding sensitivity of 0.89 (0.88–0.89), and specificity of 0.89 (0.89–0.90). Pooled sensitivity and specificity for experts endoscopists were 0.77 (0.76–0.78), and 0.92 (0.91–0.93), respectively. However, the overall SROC for the CNN model and expert endoscopists was 0.95 and 0.90. The findings of this comprehensive study show that CNN model exhibited comparable performance to endoscopists in the diagnosis of EGC using digital endoscopy images. Given its scalability, the CNN model could enhance the performance of endoscopists to correctly stratify EGC patients and reduce work load.

## 1. Introduction

Gastric cancer (GC) is the fifth most commonly diagnosed cancer and the third leading cause of death worldwide [1]. The overall incidence and global burden of GC are rapidly growing, especially in East Asian countries, such as Japan and Korea [2]. The majority of patients remain asymptomatic, and more than 80% of patients are diagnosed with GC at an advanced stage [3]. The five-year overall survival rate of GC patients at pathological stage IA is higher than 90%, where it is below 20% in stage IV [4,5]. Therefore, timely identification and referral to gastroenterologists could significantly reduce mortality and disease complications. A recent study also suggests that stratification of GC at an early stage can be clinically efficacious; although, it is quite challenging and often overlooked [6].

Importantly, previous studies showed that the detection rate of early gastric cancer (EGC) is low [7,8], and the overall false-negative rate is up to 25.8% [9,10,11,12]. Endoscopy is now a widely used technique for distinguishing between EGC and other gastric diseases (e.g., Helicobacter pylori and gastritis) [13]. Several reliable imaging modalities, namely, white light imaging (WLI) or narrow-band imaging (NBI) combined with magnifying endoscopy, have been used to clearly visualize and stratify gastric abnormalities such as cancers [14,15,16] and intestinal metaplasia [17]. A meta-analysis of 22 studies reported that the rate of missed GC when using endoscopy is only 9.4% [18]. However, grading of endoscopic images is always subjective, time-consuming, and labor intensive, and the performance varies among endoscopists, especially novices [19]. Automated grading of EGC would have enormous clinical benefits, such as increasing efficiency, accessibility, coverage, and productivity of existing resources.

**Figure 1:**
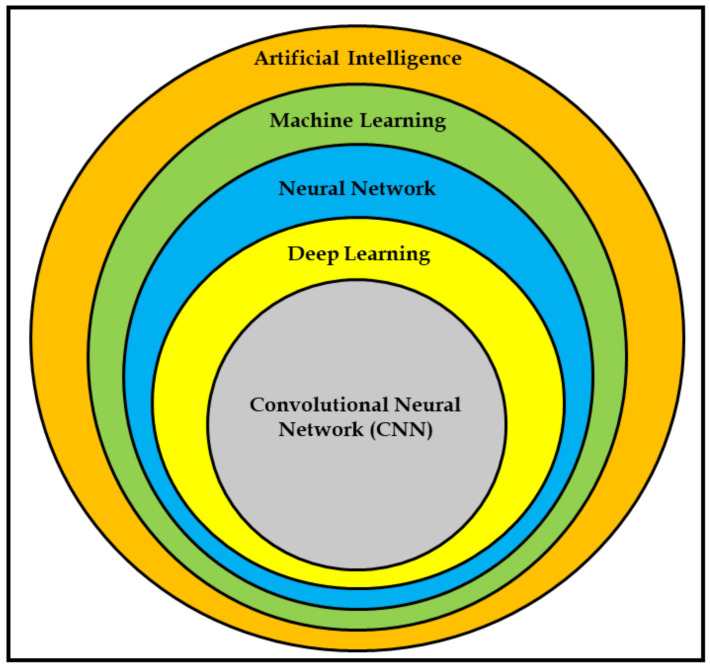
Hierarchical architecture of artificial intelligence.

Artificial intelligence (AI) has gained tremendous global attention over the last decade in various healthcare domains, including gastroenterology. AI models have shown robust performance in the diagnosis of gastroesophageal reflux disease [20] and the prediction of colorectal [21] and esophageal squamous cell carcinoma [22]. AI is a broader notion, which includes machine learning (ML) and deep learning (DL) (Figure 1). AI illustrates an innovative computerized technique to perform complex tasks that normally require “human judgement/cognition”. ML is a special branch of AI that allows a computer to become more accurate in predicting, identifying, and stratifying tasks without using explicit computer programing. ML algorithms have several potential limitations to perform tasks; primarily image recognition. However, DL, a subset of ML, has revolutionized the world and become the de-facto standard for recognizing medical images.

Recently, CNN has been applied to detect EGC using endoscopic images, helping physicians to reduce a mistaken diagnosis and improve effective clinical decisions. The primary benefits of the CNN model in gastroenterology can be to promote earlier detection, more accurate diagnosis, and ensure a more timely treatment. Developing a CNN-based automated system could detect EGC faster than endoscopists, and result in positive effects on clinical workflow and quality for patients care. However, the overall clinical applicability and reliability of the CNN model for EGC are still debated due to a lack of external validation and comparison to the performance of endoscopists. To our knowledge, there is no study that summarizes the effectiveness of the recent evidence. Therefore, the aims of this meta-analysis were to critically review the relevant articles of the CNN model for the diagnosis of EGC, evaluate the diagnostic performance in comparison with that of endoscopists, analyze the methodological quality, and explore the applicability of the CNN model in real-world clinical settings.

## 2. Materials and Methods

### 2.1. Study Protocol

We conducted a meta-analysis of studies about the diagnostic test accuracy (DTA). The methodological standards outlined for this study is based on the Handbook for DTA Reviews of Cochrane and the Preferred Reporting Items for a Systematic Review and Meta-analysis of Diagnostic Test Accuracy Studies (i.e., PRISMA-DTA), which was used to report our study findings [23].

### 2.2. Electronic Databases Search

We conducted a systematic search of electronic databases such as PubMed, Embase, Scopus, and Web of Science to identify all eligible articles published between January 1, 2010, and March 1, 2021. The following keywords were used: (1) “Deep learning” OR “Convolutional neural network” OR “CNN” OR “Artificial intelligence” OR “Automated technique”, (2) “Early gastric cancer”, (3) 1 AND 2. The reference list of potential articles was screened for other relevant studies.

### 2.3. Eligibility Criteria

We considered all studies on the diagnostic accuracy of the CNN model for detecting EGC in any setting. These original research studies were included if they were published in English, and research designs were prospective, retrospective, or secondary analyses of randomized controlled trial. We excluded studies if they were published as reviews, letters to editors, or short reports. We also excluded studies reported in invasion of GC and with a lack of DTA, namely sensitivity, and specificity. Two authors (M.M.I., T.N.P.) independently reviewed each study for eligibility and data extraction. Any disagreement during the study screening was resolved through discussion between the main investigators.

### 2.4. Data Extraction

The same two authors extracted the following data: (a) study characteristics (author first and last name, publication year, country, study design, sample size, total number of endoscopy image, and clinical settings), (b) patient characteristics (inclusion and exclusion criteria, demographic criteria), (c) index test (methods, performer of endoscopy), (d) reference standard (image modality, guidelines), and (e) diagnostic accuracy parameters (accuracy, sensitivity, specificity, and the area under the receiver operating curve).

### 2.5. Quality Assessment and Risk of Bias

The Quality Assessment of Diagnostic Accuracy Studies-2 (QUADAS-2) tool was used to assess the risk of bias of the included studies [24]. The QUDAS-2 tool contains two domains, namely risk of bias (patient selection, index test, reference standard, and flow and timing) and applicability concerns (patients’ selection, index test, and reference standard). The risk of bias was categorized into three groups, namely low, uncertain, and high.

### 2.6. Statistical Analysis

We followed the Cochrane Handbook for Systematic Reviews of Diagnostic Test Accuracy methodology guidelines to conduct all statistical analyses. The pooled sensitivity and specificity with the corresponding 95% confidence intervals (CIs) were calculated using a random-effect model. Moreover, the summary receiver operating characteristic curve (SROC) was computed by bivariate analysis. In our study, we also calculated the positive predictive value, negative predictive value, positive likelihood ratio, negative likelihood ratio, and diagnostic odd ratio. The value of the SROC curve was considered to be excellent (SROC: ≥90), good (SROC: 80–89), fair (SROC: 0.70–0.79), poor (SROC: 0.60–0.69), and worse (SROC: <50). We also assessed the statistical heterogeneity among the studies by using the I^2^ value, and the I^2^ value was also classified into very low (0–25%), low (25–50%), medium (50–75%), and high (>75%) heterogeneity, respectively [25].

## 3. Results

### 3.1. Study Selection

**Figure 2:**
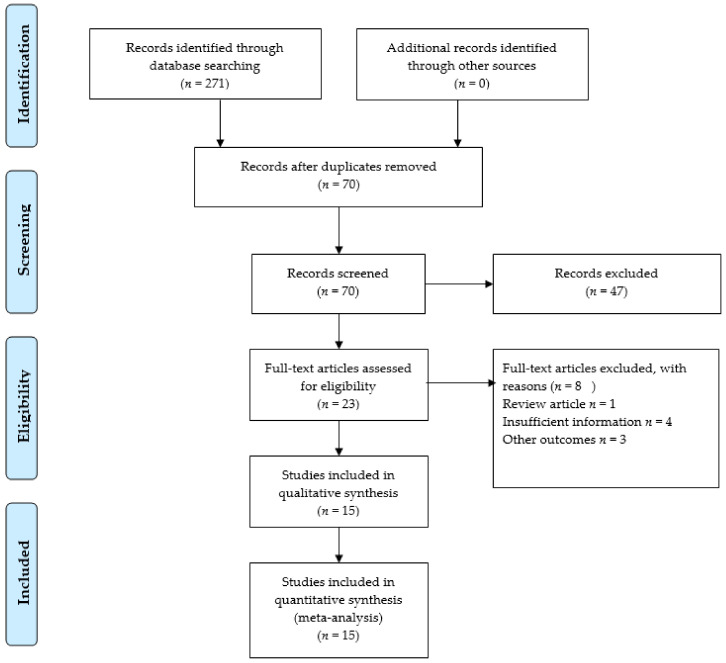
Search Strategy.

The initial literature search of the electronic databases yielded 171 articles. A total of 101 articles were excluded for duplication. After reviewing the titles and abstracts, we further excluded 47 articles; therefore, 23 articles went for full-text review. Afterwards, we screened all reference lists for further relevant articles, but no additional study was found. Based on the full-text review, we excluded eight more studies because they were not in adherence with our inclusion criteria. Finally, 15 studies met all inclusion criteria [6,26,27,28,29,30,31,32,33,34,35,36,37,38,39]. The flow diagram of the systematic search is presented in Figure 2.

### 3.2. Study Characteristics

Table 1 shows the baseline characteristics of the included studies. Among the 15 included studies, 7 studies were published in China, 6 studies in Japan, and 2 studies in Korea. All the included studies retrospectively collected data and developed their model for the diagnosis of EGC. All the studies utilized the CNN model to train and validate their results; however, GoogLeNet, Inception-v3, VGG-16, Inception-Resnet-v2, and ResNet34 were the most widely used algorithms (Table S1). The number of patients and images ranged from 69–2639 and 926–l45,240, respectively. The gold standard methods for identifying EGC were the World Health Organization (WHO) guidelines, Japanese classification, and histopathology, as shown in Table 2. White light imaging (WLI), magnifying endoscopy, narrow-band imaging (ME-NBI), and chromoendoscopy imaging were utilized to develop and evaluate the performance of the CNN model.

### 3.3. Deep Learning Model for EGC:

**Figure 3:**
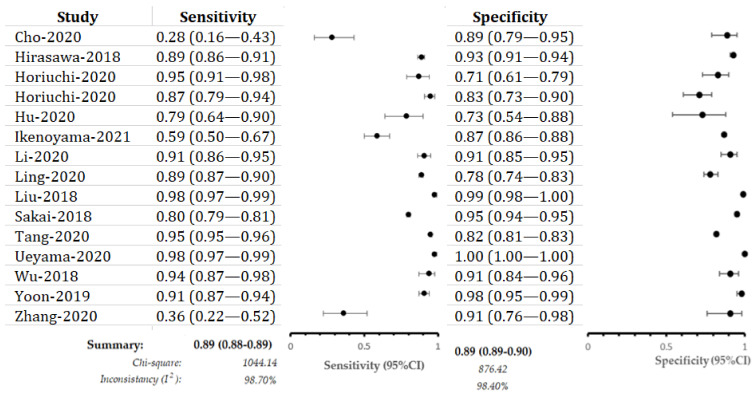
Sensitivity and specificity of included studies for EGC detection.

**Figure 4:**
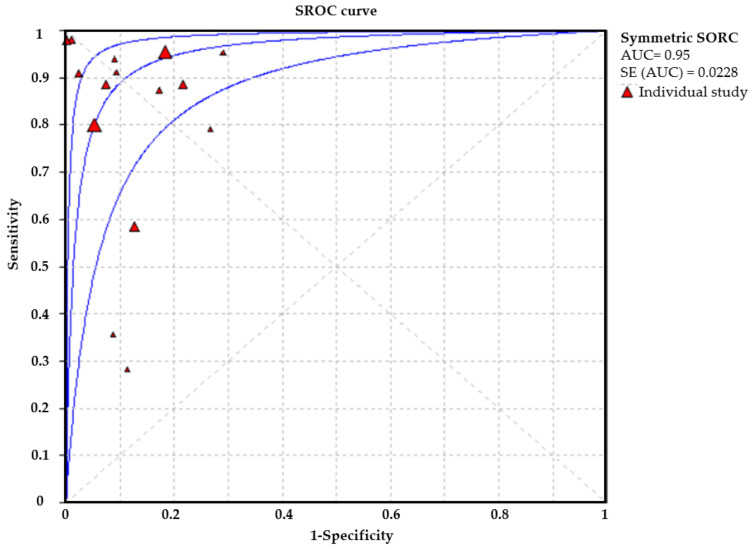
The AUROC curve for EGC detection.

A total of 15 studies focused on the performance of the CNN model for EGC detection. The pooled sensitivity was 0.89 (95%CI: 0.88–0.89), and the corresponding specificity was 0.89 (95%CI: 0.89–0.90) (Figure 3). The pooled SROC of the CNN model to detect EGC was 0.95 (Figure 4). Moreover, the pooled positive predictive value (PPV), negative predictive value (NPV), positive likelihood ratio (+LR), and negative likelihood ratio (−LR) were 0.86, 0.90, 8.44, and 0.13, respectively.

### 3.4. Performance Evaluation in Different Image Modalities

Eight studies used ME-NBI images to develop a CNN model for predicting EGC (Table 3). The pooled sensitivity and specificity of CNN model for the detection of EGC was 0.95 and 0.95, respectively. Additionally, the pooled sensitivity and specificity of WLI image application (4 studies) was 0.80 and 0.95, respectively. The performance was not up to the mark while applying mixed image for detecting EGC. The pooled sensitivity, specificity, PPV, and NPV was 0.85, 0.89, 0.63, and 0.96, respectively.

### 3.5. Deep Learning versus Endoscopists

Five studies compared the performance of the CNN model to detect EGC with a total of 51 expert endoscopists (who had more than 10 years of working experience). The pooled sensitivity, specificity, PPV, and NPV was 0.77, 0.92, 0.80, and 0.90, respectively. The pooled SROC of expert endoscopists for detecting EGC was 0.90. Five studies also compared the performance of the CNN model to detect EGC with 47 senior endoscopists (who had 5–10 years of working experience). The pooled sensitivity, specificity, PPV, and NPV was 0.73, 0.95, 0.89, and 0.84, respectively. The pooled SROC of expert endoscopists for detecting EGC was 0.92. Moreover, the pooled sensitivity, specificity, PPV, and NPV of junior endoscopists was 0.69, 0.80, 0.78, and 0.71, respectively (Table 4).

### 3.6. Quality Assessment

In this study, the risk of bias was assessed by the QUDAS-2 tool (Table S2). The risk of bias for patient’s selection, index test, and reference standard were low. All studies had an unclear risk of bias for the flow, timing, and index test. In case of applicability, all studies had a low risk of bias for the patient selection, index test, and applicability concern for the reference standard.

## 4. Discussion

### 4.1. Main Findings

This comprehensive study shows the effectiveness of the CNN model in the automatic diagnosis of EGC using endoscopic digital images. The key findings are (1) the CNN model can diagnose EGC with comparable or better performance than expert endoscopists, and (2) the CNN model may facilitate existing screening program without human efforts, avoid misclassification, and assist endoscopists when it is needed.

### 4.2. Clinical Implications

The number of GC cases and deaths has increased globally. However, the prevalence of GC is always high in developed countries (approximately 70%), and nearly 50% of GC occurred in East Asian countries such as China, Korea, Japan, and Taiwan [40,41]. Previous studies reported that earlier identification and treatment could reduce the overall morbidity and mortality of GC [19,42]. Patients with gastrointestinal disorders such as Helicobacter pylori, gastritis, and intestinal metaplasia should be screened for GC at least annually to identify high-risk patients. In practice, the screening strategy relies only on visual inspection of the gastric mucosa [43]; therefore, gastroenterologists use an endoscope to collect samples from the inner cavity for histopathological evaluation [44]. Endoscopy is considered as a standard procedure for the diagnosis of EGC, and detection is higher than other screening methods such as UGI series, serum pepsinogen testing, and H. pylori serology [45]. However, the use of endoscopic screening has several limitations, and screening requires referral to a gastroenterologist. Patients do not always visit expert gastroenterologists due to the logistical barrier, cost, and availability of experts in rural areas [46].

Moreover, manual inspection of endoscopy images for gastric abnormalities findings is time-consuming, and detection performance always depends on the skill of the endoscopists. Previous studies reported that manual inspection increases the false detection rate, especially when the number of patients for screening is high [47,48]. Our study findings demonstrate that the CNN model can improve the detection performance of EGC, which is higher than that of endoscopists. Tang et al. [35] reported that the detection performance of EGC is even higher when endoscopists use the CNN model (Table 4). Obtaining high-quality images to detect EGC is difficult, especially for inexperienced endoscopists. Different image techniques have been using to detect gastric tissue abnormalities. However, the CNN model, which used a conventional technique, white light endoscopy (WLE), had lower performance NBI, a novel imaging technique. A previous study mentioned that diagnosis accuracy of EGC when using WLE is low when it comes to flat lesions and minute carcinoma [49]; however, both superficial structures and microvascular architecture of lesions are visualized by NBI [50,51]. The performance of CNN was even lower when a mixture of WLI, ME-NBI, and chromoendoscopy had been used to train and test the model.

**Figure 5:**
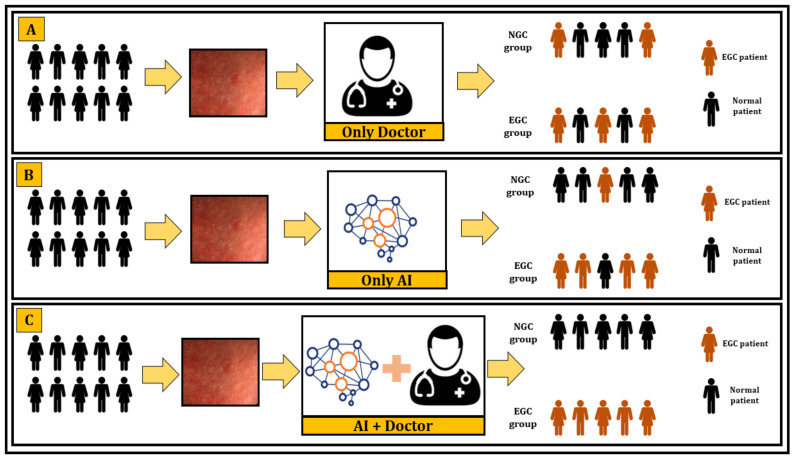
Propose diagnosis of EGC by man with machine. (**A**) Screening of EGC by physicians only can increase false-positive and false-negative cases; (**B**) screening of EGC by AI only can also increase false-positive and false-negative; (**C**) combined decision based on AI plus physicians can accurately diagnose EGC.

The findings of our study suggest that the CNN model is clinically effective in detecting EGC. The application of the CNN model to correctly diagnose EGC could provide alternative ways for EGC screening, especially in areas where skilled endoscopists are not always available. In the future, physicians may cooperate with a CNN-based automated system, which would help to increase work efficiency and to reduce false detection (Figure 5).

### 4.3. Strengths and Limitations

Our study has several strengths. First, this is the most comprehensive study that evaluated the performance of the CNN model to correctly diagnose EGC. Second, our study also compared the performance of the CNN model with that of expert, senior, and junior endoscopists to diagnose EGC, which has great clinical value. Third, we also compared the performance of the CNN model for different image modalities. Finally, we calculated the overall PPV and NPV values, which may help to make an effective clinical decision on implementing the CNN model in real-world clinical settings. However, our study has several limitations that also need to be mentioned. First, our study findings are mainly based on retrospective data, but prospective evaluation is needed to check the real performance of the CNN model. Although, several studies had prospective evaluation. Second, all studies used high-quality images to develop and validate the performance of the CNN model. Therefore, our study is unable to present the real-performance of the CNN model if subjected to lower quality images. Finally, high heterogeneity exists among the studies included in this current study, which may be due to the following reasons: (a) varied nature of methodology and training algorithms, (b) a different number of sample size, (c) the variability of endoscopic images (WLI, NBI, and chromo-endoscopy). However, it could also be due to the distinct strictness of experts in the various study centers for positive judgment of GC patients. Therefore, the findings should be interpreted with caution. Despite the above limitations, efforts were made to select high-quality studies and the current meta-analysis presents the potentiality of the DL model for detecting GC. These findings warrant further validation in the larger prospective studies with different populations.

## 5. Conclusions

This study provides a summary of the current state-of-the-art CNN model for the diagnosis of EGC using endoscopic images. The findings of this comprehensive study show that the CNN model had a high sensitivity and specificity of stratifying EGC and outperformed the performance of endoscopists. A fully automated tool based on CNN could facilitate EGC screening in a cost-effective and time-efficient manner.

Despite the outstanding performance of the CNN model, there are still several potential challenges to apply these findings in the real-world clinical practice. First, the CNN model is often referred to as “black-box” due to a lack of interpretability of its findings [52,53,54,55]; therefore, it is not sufficient to have good accuracy. Second, the comparison of CNN algorithms across the studies is quite challenging because various methodologies on different populations with different sample sizes were being compared. Third, more sample size, and sample from various population as developing set is likely to improve performance, reduce the risk of bias, and increase the applicability of DL models in the real-world clinical settings. Finally, generalizability is another key challenge because the performance of the CNN model could vary when it is tested on unknown datasets, especially those based on low-quality images. Therefore, more evaluation is needed before widely deploying the CNN based tool in the real-world clinical practice.

## Figures and Tables

**Table 1:** Baseline characteristics of included studies.

Study	Country	Year	Design	Model (Algorithm)	Total Images	Total Patients	Data Partition Process	External Validation	Sen/Spe	Level
Cho-2020 [26]	Korea	2010–2017	Retrospective	CNN (Inception-Resnet-v2)	5017	200	Split	Yes	0.283/0.883	AGC, EGC, HGD, LGD, and non-neoplasm
Hirasawa-2018 [27]	Japan	2004–2016	Retrospective	CNN (SSD)	2296	69	Split	No	0.885/0.927	EGC, NGC
Horiuchi-2020 [28]	Japan	2005–2016	Retrospective	CNN (GoogLeNet)	2570	NR	Split	No	0.954/0.710	EGC, gastritis
Horiuchi-2020 [29]	Japan	2005–2016	Retrospective	CNN (GoogLeNet)	2570	82	Split	No	0.874/0.828	EGC, NGC
Hu-2020 [30]	China	2017–2020	Retrospective	CNN (VGG-19)	1777	295	Split	Yes	0.792/0.745	NN, MLGN, LC, SIC, EGC
Ikenoyama-2021 [6]	Japan	2004–2016	Retrospective	CNN (SSD)	13,584	2639	Split	No	0.59/0.87	EGC, NGC
Yoon-2019 [38]	Korea	2012–2018	Retrospective	CNN (VGG-16)	11,539	800	Split	No	0.910/0.976	EGC, NGC
Li-2019 [31]	China	2017–2018	Retrospective	CNN (Inception-v3)	10,000	NR	Split	No	0.9118/0.906	EGC, NGC
Ling-2020 [32]	China	2015–2020	Retrospective	CNN (VGG-16)	9025	561	Split	Yes	0.886/0.786	EGC, NGC
Liu-2018 [33]	China	NR	Retrospective	CNN (Inception-v3)	2331	NR	Split	No	0.981/0.988	EGC, NGC
Sakai-2018 [34]	Japan	NR	Retrospective	CNN (GoogLeNet)	926	58	Split	No	0.800/0.948	EGC, NGC
Tang-2020 [35]	China	2016–2019	Retrospective	CNN (DCNN)	l45,240	1364	Split	Yes	0.955/0.817	EGC, NGC
Ueyama-2020 [36]	Japan	2013–2018	Retrospective	CNN (ResNet50)	5574	349	Split	No	0.98/1.0	EGC, NGC
Wu-2018 [37]	China	2016–2018	Retrospective	CNN (VGG-16+ResNet50)	NR	NR	Split	Yes	0.940/0.910	EGC, NGC
Zhang-2020 [39]	China	2012–2018	Retrospective	CNN (ResNet34)	21,217	1121	Split	No	0.360/0.910	EGC, NGC

**Table 2:** Description of endoscopy and images.

Study	Data Source	Format	Rotation	Resolutio	Level of Annotator Experience	Gold Standard	Image Terminology	Endoscope
Cho-2020	Two Hospitals(CHH & DTSHH)	JPEG	35-field view	1280 * 640	Expert	Histopathology	WL	GIF-Q260, H260 or H290, CV-260 SL or Elite CV-290
Hirasawa-2018	Two Hospitals (CIH & TTH); Two Clinics (TTIG & LYC)	NR	NR	300 * 300	Expert	Japanese classification	WL, ME-NBI, Chromoendoscopy	GIF-H290Z, GIF-H290, GIF-XP290N, GIF-H260Z, GIF-Q260NS, EVIS LUCERA CV-260/CLV-260 EVIS LUCERA ELITE CV-290/ CLV-290SL
Horiuchi-2020	Single Center (CIH)	NR	NR	224 * 224	Expert	Histopathology	ME-NBI	GIF-H260Z and GIF-H290Z
Horiuchi-2020.	Single Center (CIH)	NR	NR	224 * 224	Expert	Histopathology	ME-NBI	GIF-H240Z, GIF-H260Z, and GIF-H290Z:
Hu-2020	Single Center (ZH)	NR	NR	224 * 224	Expert	Histopathology	ME-NBI	GIF-H260Z or GIF-H290Z
Ikenoyama-2021	Single Center (CIH)	NR	Anterograde & retroflexed view	300 * 300	Expert	Histopathology	WL, NBI, Chromoendoscopy	GIF-H290Z, GIF-H290, GIF-XP290N, GIF-H260Z, GIF-Q260J, GIF-XP260, GIF-XP260NS, GIF-N260
Yoon-2019	Single Hospital (GSH)	NR	both close-up and a distant view	NR	Expert	WHO classification of tumor & Japanese classification	WL	GIF-Q260J, GIF-H260; GIF-H290
Li-2019	Four Hospitals	NR	NR	512 * 512	Expert	Vienna classification	ME-NBI	GIF-H260Z; GIF-H290Z
Ling-2020	Renmin Hospital	NR	NR	512 * 512	Expert	Japanese classification	ME-NBI	GIF-H260Z
Liu-2018	Chongqing Xinqiao Hospital	JPEG	Horizontally, and vertically	768 * 576, 720 * 480, 1920 * 1080, 1280 * 720	Expert	NR	ME-NBI	GIF Q140Z; GIF-H260Z
Sakai-2018	NR	NR	NR	224 * 224	Expert	Histopathology	WL	GIF-H290Z; GIF TYPE H260Z
Tang-2020	Multi-center	NR	NR	NR	Expert	WHO classification; Japanese classification; European society of gastrointestinal endoscopy	ME-NBI	GIF-H260, GIF-H260Z, GIFHQ290, GIF-H290Z, EVIS LUCERA CV260/CLV260SL, EVIS LUCERA ELITECV290/CLV290SL
Ueyama-2020	Saitama Medical Center	NR	NR	224 * 224	Expert	Japanese classification	ME-NBI	(GIF-H260Z; GIF-H290Z
Wu-2018	Renmin Hospital	NR	NR	224 * 224	Expert	Histopathology	WL, ME-NBI	CVL-290SL, VP-4450HD
Zhang-2020	Peking University People’s Hospital	NR	NR	NR	Expert	Japanese classification	WL	GIF-H260, GIF-Q260J, GIF-H290, EVIS LUCERA CV-260/CLV-260

Note: CHH and DTSHH; CIHA: Cancer Institute Hospital Ariake, Tokyo, Japan; TTH: Tokatsu-Tsujinaka Hospital, Chiba, Japan; TTIGP: Tada Tomohiro Institute of Gastroenterology and Proctology, Saitama, Japan; LYC: Lalaport Yokohama Clinic, Kanagawa, Japan); CIH: Cancer Institute Hospital; ZH = Zhongshan Hospital; GSH: the Gangnam Severance Hospital; SYSUCC: Sun Yat-sen University Cancer Center, Guangzhou, China; NR = Not reported. *: Multiple sign.

**Table 3:** The performance of the CNN model for EGC detection in different image modalities.

Model	SROC	SN	SP	PPV	NPV	+LR	−LR	DOR
CNN_WLI_	0.99	0.80	0.95	0.94	0.83	9.32	0.33	28.47
CNN_ME−NBI_	0.97	0.95	0.85	0.87	0.93	7.84	0.07	123.45
CNN_WLI+ME−NBI+C_	0.96	0.85	0.89	0.63	0.96	8.27	0.16	51.44

Note: SN: Sensitivity; SP: Specificity; PPV: Positive Predictive Value; NPV: Negative Predictive Value; +LR: Positive Likelihood Ratio; −LR: Negative Likelihood Ratio; WLI: White Light image; ME-NBI: Magnifying endoscopy with narrow-band imaging; C: Chromoendoscopy.

**Table 4:** Comparison between deep learning and endoscopists.

Comparison	SROC	SN	SP	PPV	NPV	+LR	−LR	DOR
CNN	0.95	0.86	0.89	0.87	0.87	10.00	0.13	75.17
Experts	0.90	0.77	0.92	0.80	0.90	5.84	0.22	27.99
Seniors	0.92	0.73	0.95	0.89	0.84	7.90	0.24	33.88
Junior	0.82	0.69	0.80	0.78	0.71	3.83	0.36	11.09
CNN + Expert †	-	0.97	0.91	0.91	0.98	-	-	-
CNN + Junior †	-	0.94	0.97	0.98	0.95	-	-	-

Note: CNN: Convolutional Neural Network; †: reported only Tang et al.; Experts: had more than 10 years’ experience; seniors: had 5–10 years’ experience; junior: had less than 5 years’ experience.

## Data Availability

The data presented in this study are available on request from the corresponding author.

## Supplementary Materials

The following are available online at https://www.mdpi.com/article/10.3390/cancers13215253/s1, Table S1: Description of performance metrics, data and model description, Table S2: Quality Assessment of Diagnostic Accuracy Studies-2 for Included Studies.
