# Supplementary material for: Artificial Intelligence in Gastric Cancer: Identifying Gastric Cancer Using Endoscopic Images with Convolutional Neural Network
Source: Cancers (Basel). 2021 Oct 20;13(21):5253. doi: 10.3390/cancers13215253 (PMC8582393; doi:10.3390/cancers13215253)
Supplement: Supplementary file 1 [file cancers-13-05253-s001.zip › cancers-1404132-supplementary.pdf]

Supplementary Materials: Artificial Intelligence in Gastric Cancer: Identifying Gastric Cancer Using Endoscopic Images with Convolutional Neural Network

Md. Mohaimenul Islam [1,2,3], Tahmina Nasrin Poly [1,2,3], Bruno Andreas Walther [4], Ming-Chin Lin [1,5,6] and Yu-Chuan (Jack) Li [1,2,3,*]

Table S1. Description of performance metrics, data and model description.

Study	Accuracy	AUROC	Data Availability	Code Availability	Data Preprocessing	Process of Transfer Learning
Cho-2020	74.5	58.3	No	No	Excluded the image with poor quality or low resolutions. They also excluded the images from image-enhance endoscopy and image without pathology results. Pathological diagnosis of endoscopic biopsy was made by two specialist pathologists, each with more than 10 years of experience.	A stochastic approximation of gradient descent optimization was done with the Adam optimizer. The initial learning rate, end learning rate, weight decay, and batch size were 0.01, 0.0001, 5e-05, and 30, respectively. The training dataset was preprocessed to enhance recognition performance by random cropping, resizing, flipping, and color adjustments implemented internally in each CNN model.
Hirasawa-2018	-	-	No	No	Excluded the images if they were magnified as well as poor quality images resulting from less insufation of air, post-biopsy bleeding, halation, blur, defocus, or mucus. At least one gastric cancer lesion was presented in all images, and multiple images were prepared for a same lesion to include	CNN layers were fne-tuned using stochastic gradient descent with a global learning rate of 0.0001. Each image was resized to 300 × 300 pixels, and the bounding box was also resized accordingly to make CNN analyze optimally.

					diferences in angle, distance, and extension of the gastric wall.	
Horiuchi-2020	85.3	-	No	No	Excluded images with mucus and blood adhering extensively; images in which EGC or gastritis was difficult to evaluate because of inadequate focus or halation; in extracted gastritis images, to exclude tumor refection, cases where tumorous lesions were present in the stomach.	The CNN system was trained using backpropagation, a method of training neural networks, by which loss gradients for all weights in the network can be computed efficiently. To optimize images for GoogLeNet, they were resized to 224×224 pixels. All layers were tuned by adam.
Horiuchi-2020.	85.1	-	No	No	Excluded images and videos with mucus, excessive blood adherence, inadequate focus, or halation; and only noncancerous images and videos that included some EGC.	All network layers were finetuned using Adam, 22 a method for stochastic optimization with a global learning rate of 0.0001 and batch size of 32. To optimize our images for GoogLeNet, we resized them to 224 224 pixels.
Hu-2020	0.77	0.80	No	No	Images were excluded if (1) the patient had multiple lesions; (2) had no definitive pathological diagnosis; and (3) images were too blurry to be evaluated by senior endoscopists due to inadequate focus, halation, excessive bubbles, or bleeding. Two experienced pathologists were blinded to the magnified endoscopic findings performed a histological evaluation according standard guidelines.	All image were resized to 224×224 pixels to match the model. For each epoch, every 16 images were divided as a batch to be placed into the model. To optimize the model, the cross-entropy loss was employed during the training process. Adam optimizer with an initial learning rate of 10-6 and a weight decay of 0.01 was used.

Ikenoyama-2021	--	75.7	No	No	Images containing poor insufflation, post-biopsy bleeding, halation, blur, defocus, or mucus were excluded from the training Dataset.	All EGC lesion were confirmed histologically using biopsy. All gastric cancer lesions in the training dataset were manually annotated with rectangular bounding boxes by an expert endoscopist.
Yoon-2019	--	98.1	No	No	The image of the lesion should have both close-up and a distant view so that the size and position of the lesion can be identified. Additionally, the amount of gas insufflation should be adjusted appropriately to reflect the condition of the lesion and its surrounding area. Poor quality images were excluded.	The first model was a typical method that computed the loss between the real and predicted classes of input data. The second was a novel method that used the weighted sum of gradient-weighted class activation mapping (Grad-CAM) and cross-entropy losses. To let the model focus on the fine-grained features of EGC regions, we employed a novel loss function by adding Grad-CAM.
Li-2019	90.91	-	No	No	Expert's endoscopists (more than ten years experience) retrospectively diagnosed gastric mucosal lesions. Two pathologists also perform histological evaluation based on neoplasm guidelines. Due to the imbalance of sample size, they increased the sample size using image processing methods such as image rotate transform, image mirror transform,	Four steps: a) create model and load the pre-trained inception-v3 model to initialize the model weight, b) remove the top layer and place full-connected layer with the output value of 2 outcome c) fine-tuning, and d) accelerate the convergence of the model.

					image cut, image brightness variations, image blur, and up-sampling	
Ling-2020	86.2	-	No	No	Images of poor quality (resulted from defocus, halation, blurs and so on) were excluded by two doctorial students, and then the expert endoscopist reviewed and removed images of noncancerous background mucosa.	Replaced the final classification layer with another fully connected layer using transfer learning, retrained them using our datasets, and fine-tuned the parameters to fit our needs. The dataset was randomly divided into 5 subsets.
Liu-2018	98.5	-	-	-	-	-
Sakai-2018	87.6	-	No	No	The identified lesions were translated into binary images and used as the ground truth. Cropped approximately 100 images of size 224 × 224 pixels randomly from each of the 100 cancer images that were selected from the 228 cancer images. Augmentations, including rotation, shift, shear, zoom, and flip were also applied.	For the fine-tuning, we trained the initial network for 50 epochs with learning rates of 0.0001 and 0.00001 before and after 34 epochs, respectively. The network parameters were optimized using stochastic gradient descent with a mini-batch size of 32.
Tang-2020	-	94.0	http://112.74.182.39/	No	Images of NBI, dye-stained imaging, ESD operation, or poor quality (e.g., less insufflation of air, halation, defocus, blurs, bubbles, sliding, fuzzy, bleeding) were excluded. Two board-certified pathologists determined the pathologic diagnosis of EGC using haematoxylin- and eosin-stained tis-	The backbone structure employed in this model was the Darknet-53 model, which contains 53 layers of neurones. The architecture consists of a sequence of nonlinear processing modules.

					sue slides, according standard guidelines.	
Ueyama-2020	98.7	-	No	No	ME-NBI images of gastric adenocarcinoma of fundic gland-type (GAFG) and diffuse-type EGC, as well as unanalyzable and low-quality images, were excluded.	replaced the final classification layer with another fully connected layer, retrained it using our training dataset, and fine-tuned the parameters of all layers. Images were resized to 224 × 224 pixels to suit the original dimensions of the models. A rotation augmentation was also applied to images.
Wu-2018	92.5	-	No	No	Extensive attention was paid to ensure that images from the same person were not split between the training, validation, and test sets	replaced the final classification layer with another fully connected layer, retrained it using our datasets, and fine-tuned the parameters of all layers. Images were resized to 224 × 224 pixels to suit the original dimensions of the models.
Zhang-2020	78.7	-	No	No	Magnified endoscopy (ME) images; chromoendoscopy images, enhanced endoscopy images, blurry images, and images stained with mucus, blood, etc were excluded. Two endoscopists with over ten years of experience each in gastroscopy diagnosis labeled all the images, carefully marking the margins of the lesions with lines and completing the information	The initial weight for ResNet34 is obtained by using transfer learning for pretraining on ImageNet. The weight is subsequently fine-tuned using the random gradient descent method, with an initial learning rate of 0.0001 and a batch size of 32

		concerning the lesion in a dialog box, including the lesion size, the depth of invasion, and the pathology.	

Table S2. Quality Assessment of Diagnostic Accuracy Studies-2 for Included Studies.

Study	Risk of Bias				Applicability Concerns		
	Patients Selection	Index Test	Reference Standard	Flow and Timing	Patients Selection	Index Test	Reference Standard
Cho-2020	☺	☺	☺	?	☺	☺	☺
Hirasawa-2018	☺	☺	☺	?	☺	☺	☺
Horiuchi-2020	☺	☺	☺	?	☺	☺	☺
Horiuchi-2020.	☺	☺	☺	?	☺	☺	☺
Hu-2020	☺	☺	☺	?	☺	☺	☺
Ikenoyama-2021	☺	☺	☺	?	☺	☺	☺
Yoon-2019	☺	☺	☺	?	☺	☺	☺
Li-2019	☺	☺	☺	?	☺	☺	☺
Ling-2020	☺	☺	☺	?	☺	☺	☺
Liu-2018	☺	☺	☺	?	☺	☺	☺
Sakai-2018	☺	☺	☺	?	☺	☺	☺
Tang-2020	☺	☺	☺	?	☺	☺	☺
Ueyama-2020	☺	☺	☺	?	☺	☺	☺
Wu-2018	☺	☺	☺	?	☺	☺	☺
Zhang-2020	☺	☺	☺	?	☺	☺	☺

☺= low risk, ? = Uncertain
